# Characteristics of fever and response to antipyretic therapy in military personnel with adenovirus-positive community-acquired pneumonia

**DOI:** 10.1186/s40779-020-00235-x

**Published:** 2020-02-21

**Authors:** Hongseok Yoo, Jimi Oh, Chul Park

**Affiliations:** 1 Division of Pulmonary and Critical Care Medicine, Department of Internal Medicine, Samsung Medical Center, Sungkyunkwan University School of Medicine, Seoul, 06351 South Korea; 2 Department of Anesthesiology and Pain Medicine, Asan Medical Center, University of Ulsan College of Medicine, Seoul, 05505 South Korea; 3 Division of Pulmonary Medicine, Department of Internal Medicine, Wonkwang University Hospital, Iksan, 54538 South Korea

**Keywords:** Adenovirus, Fever, Response to antipyretic treatment

## Abstract

**Background:**

In 2014, an outbreak of adenoviral pneumonia occurred in the Korean military training center. However, there are limited data on the characteristics of the fever and its response to antipyretic therapy in immunocompetent adults with adenovirus-positive community-acquired pneumonia (CAP).

**Methods:**

The medical records of the patients who were admitted to the Armed Forces Chuncheon Hospital for the treatment of CAP between January 2014 and December 2016 were retrospectively analyzed. The patients were divided into three groups, namely, the adenovirus-positive (Adv) group, the adenovirus-negative (Non-Adv) group and the unknown pathogen group, according to the results of a polymerase chain reaction (PCR) test and sputum culture used to measure adenovirus and other bacteria or viruses in respiratory specimens. We evaluated and compared the demographics, clinicolaboratory findings and radiological findings upon admission between the two groups.

**Results:**

Out of the 251 military personnel with CAP during the study periods, 67 were classified into the Adv group, while 134 were classified into the Non-Adv group and 50 were classified into the unknown pathogen group. The patients in the Adv group had a longer duration of fever after admission (3.2 ± 1.6 *vs.* 1.9 ± 1.2 *vs.* 2.2 ± 1.5 days, *P* = 0.018) and symptom onset (5.8 ± 2.2 *vs.* 3.9 ± 2.5 *vs.* 3.7 ± 2.0 days, *P* = 0.006) than patients in the Non-Adv and unknown pathogen groups, respectively. The patients in the Adv group had a higher mean temperature at admission (37.8 ± 0.3 *vs.* 37.3 ± 0.3 *vs.* 37.3 ± 0.3, *P* = 0.005), and more patients were observed over 40 and 39 to 40(14.9% *vs.* 2.2%* vs.* 4.0%, 35.8% *vs.* 3.7% *vs.* 6.0%, *P* <  0.001) than those in the Non-Adv and unknown pathogen groups, respectively. The Adv group more commonly had no response or exhibited adverse events after antipyretic treatment compared to the Non-Adv group (17.9% *vs.* 1.5%, 35.0% *vs.* 4.3%, *P* <  0.001, *P* = 0.05, respectively). In addition, the time from admission to overall clinical stabilization was significantly longer in the patients in the Adv group than in those in the Non-Adv group (4.3 ± 2.8 *vs.* 2.9 ± 1.8 days, *P* = 0.034, respectively). Furthermore, no significant difference in the length of hospital stay was observed between the two groups, and no patient died in either group.

**Conclusion:**

In this study, Adv-positive CAP in immunocompetent military personnel patients had distinct fever characteristics and responses to antipyretic treatment.

## Background

Adenoviruses (Adv) are nonenveloped, double-stranded DNA viruses that can cause upper and lower respiratory tract infections either in sporadic fashion or as epidemics. Currently, 49 distinct Adv serotypes have been isolated from humans. Adv typically cause mild self-limited respiratory infections. Although Adv can cause a variety of clinical manifestations, in immunocompromised patients, adenovirus infection often leads to fatal outcomes. For example, in immunodeficiency states such as a solid organ or stem cell transplantation, severe adenovirus infection may occur, with mortality up to 80% [1–4].

Outbreaks of adenoviral pneumonia have been occasionally reported in young adult women or military personnel [5–9]. Respiratory tract infection is the leading cause of hospitalization of military trainees in the medical field. A study by the US military showed that 10% of recruits at boot camp were infected with Adv, and 90% of patients with pneumonia were infected with adenovirus [10, 11]. In 2006, a study by the South Korean military reported that the prevalence of Adv was 61% among military recruits with acute respiratory symptoms [12–16]. Upper respiratory tract infections caused by Adv may also progress to pneumonia. Recently, several case series were reported describing recruits that died in boot camp due to severe adenoviral pneumonia in South Korea since 2012 [13, 15–17]. Even if mortality results or clinical outcome of Adv infection could be affected by selection bias, it has been reported that Adv infection may be severe with a higher incidence of progression to respiratory and multiorgan failure compared to other viral etiologies with the exception of influenza. Thus, some clinical data have been reported regarding predictive factors of respiratory failure associated with Adv infection [18, 19].

If Adv infection can be diagnosed early, increased monitoring and early applied organ support may improve the clinical outcome of these patients. However, sufficient data on the distinctive characteristics of Adv infections in immunocompetent patients is currently unavailable. We previously observed that adenovirus-positive community-acquired pneumonia (CAP) patients have a high fever and respond differently to antipyretic treatment compared to CAP patients who test positive for other viruses or bacteria. Therefore, the primary goal of this study was to compare the clinical characteristics of Adv- and Non-Adv-positive CAP patients among immunocompetent military personnel and to identify the distinctive characteristics.

## Methods

### Study design and definition

This study was a single-center, retrospective cohort study. We reviewed the medical records of patients who were admitted to the Armed Forces Chuncheon Hospital (Gangwon Province, South Korea, the Referral Hospital for Gangwon Province) for CAP treatment between January of 2014 and December of 2016. Based on the unique characteristics of the Korean military medical system, all military personnel was treated initially in the military hospital despite a lack of diagnostic modalities. Ethical approval was obtained from the Institutional Review Board of the Armed Forces Medical Command (AFMC-16051-IRB-16-041), which waived the need for informed consent because of the retrospective observational nature of the study.

The patients were included in this study when they 1) were admitted for CAP treatment; and 2) had viral polymerase chain reaction (PCR) tests performed on the upper respiratory specimen. The exclusion criteria were as follows: 1) a respiratory viral PCR test was not performed; 2) they had incomplete records; 3) they were immediately transferred to a tertiary hospital for advanced care; and 4) the primary reason for admission was to manage comorbid diseases.

CAP was defined using the definition set forth by the Infectious Society of America/American Thoracic Society Consensus Guidelines [20]. In short, CAP was diagnosed when the patients had symptoms associated with respiratory tract infections and had new-onset lung infiltration or pleural effusion on chest X-rays or chest computed tomography scans. We defined fever as any temperature greater than or equal to 38 °C recorded by the tympanic route. The body temperatures of all patients were checked every hour at admission day. During the days after admission, the body temperature was often measured within 1 h when patients had a febrile sense or worsening signs of inflammation. We also recorded body temperature at the beginning of antipyretics administration. There is no standardized antipyretic treatment protocol for fever control. In our study, antipyretic therapy was administered upon reaching a body temperature ≥ of 38 °C. The interval of antipyretics administration was according to pharmacodynamics, although we performed additional antipyretics treatment when patients had a fever (two consecutive measurements ≥38 °C) and deterioration of clinical symptoms, including myalgia, general weakness, cough, nasal congestion, or dyspnea within 6 h after antipyretics administration. Responsiveness to antipyretic treatment was classified as follows: a complete response was recorded when a body temperature drop below 38 °C was observed after antipyretics treatment and sustained throughout; a partial response was recorded when a body temperature drop below 38 °C was observed but there was a resurge during observation or a need for additional antipyretics; and no response was recorded when a sustained body temperature above or equal to 38 °C was observed after antipyretics use. Unresponsive to initial antibiotic treatment was defined as observed deterioration as evidenced by worsening of clinical symptoms and signs and/or progression of lesions on radiological studies after 48 to 72 h of initial antibiotics treatment.

### Data collection and patient management

All data, including age, sex, smoking history, comorbid conditions, symptoms and clinical signs, initial laboratory and radiological findings, culture results, pneumonia severity index, clinical course, length of hospital stay, and survival outcome were collected from electronic medical records. We evaluated etiologies by sputum, nasopharyngeal or oropharyngeal secretions, blood, and urine using a microbiological culturing approach. Respiratory specimens were typically obtained from self-extracting sputum. When sputum specimens could not be obtained, upper respiratory tract specimens, such as oropharyngeal or nasopharyngeal swabs were used for viral PCR tests. Multiplex real-time PCR was performed using a Real-Q RV Detection kit (BioSewoom, Seoul, Korea) with a Roche Light Cycler 480 II instrument (Roche Diagnostics, Mannheim, Germany). Respiratory viruses included in this test are as follows; adenovirus, rhinovirus, influenza virus A/B, respiratory syncytial virus A/B, metapneumovirus, bocavirus, coronavirus, and parainfluenza virus 1/2/3. All patients were given chest X-rays and/or high resolution computed tomography (HRCT) at the time of our emergency department visit.

Initial antibiotic agents were intravenously administered to all of the patients. Initial antibiotic regimens were followed by adherence to the “Treatment Guidelines for Community-acquired Pneumonia in Korea: An Evidence-based Approach to Appropriate Antimicrobial Therapy” from The Korean Academy of Tuberculosis and Respiratory Diseases [21]. The antipyretic agents and regimens used in this study were as follows. Propacetamol was intravenously administered at a dose of 1 to 2 g as needed to a maximum of 8 g per day. Acetaminophen was given orally at a dose of 2 tablets (650 mg per tablet) every 8 h to a maximum of 6 tablets per day. Physical cooling methods applied to all febrile patients included external air, ice bag, or water blanket techniques.

### Statistical analysis

The data are presented as the means ± standard deviation or as the median [interquartile range] for continuous variables and as numbers and percentages for categorical variables. The data were analyzed using Kolmogorov-Smirnov tests for normal distribution. The data were compared using the Mann-Whitney *U*-test or Student’s *t*-test for continuous variables and the *χ*^*2*^ or Fisher’s exact test for categorical variables. Statistical analyses were performed using SPSS version 23.0 (SPSS Inc., Chicago, IL, USA), and a two-sided *P*-value < 0.05 was considered to indicate significance.

## Results

### Study participants

**Fig. 1:**
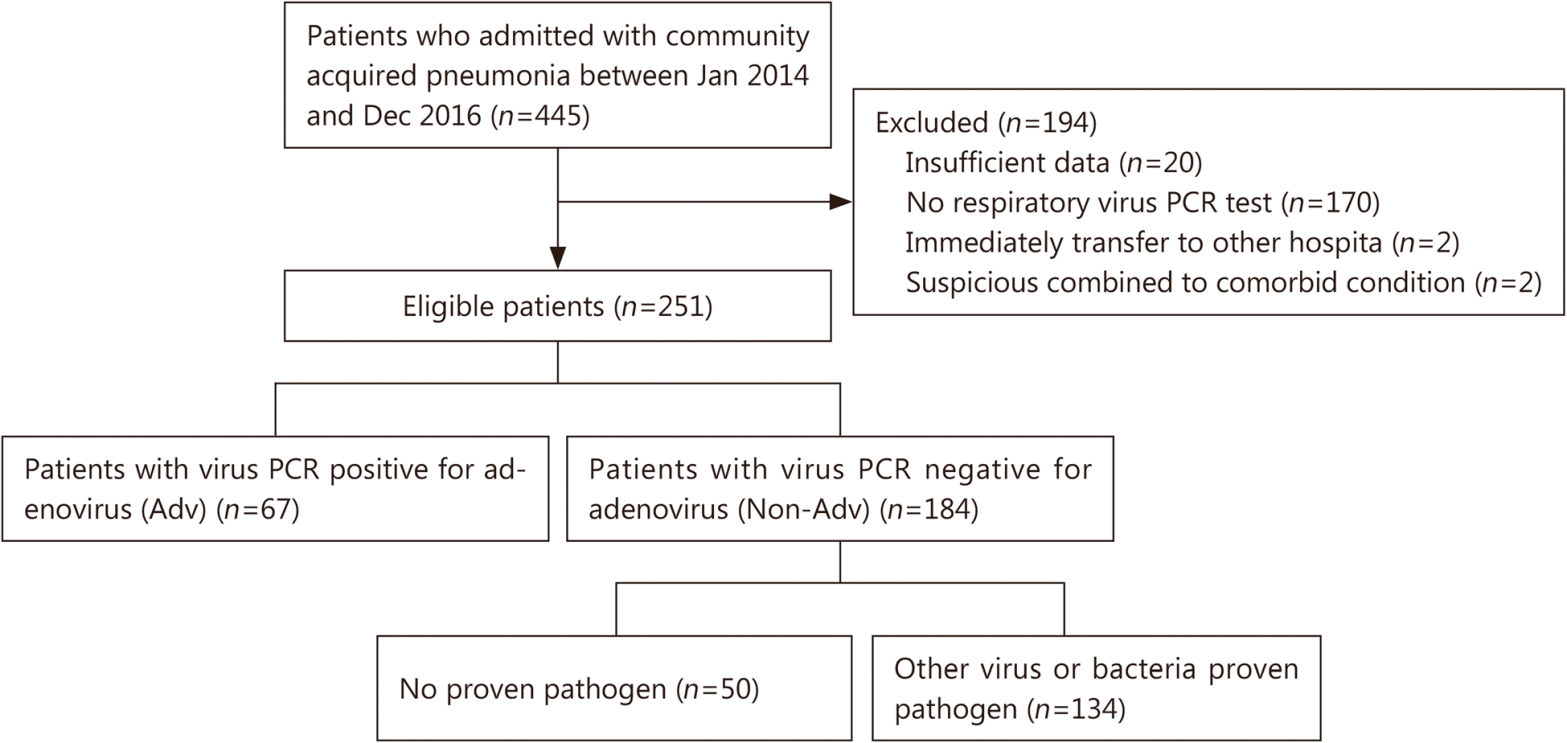
Study flow diagram

During the study period, there were 445 CAP patients admitted to the Armed Forces Chuncheon Hospital (Fig. 1). All patients were admitted via the emergency department. Out of 445 cases, 194 cases were excluded. The reasons for exclusion were no respiratory viral PCR test for 170 patients, incomplete data for 20 patients, immediately transferred to the tertiary medical center for two patients, and admission for treatment of underlying diseases for two patients. Two patients who were admitted suspicious combined to underlying disease managed to acute asthma exacerbation. Consequently, 251 patients were enrolled in this study, among whom 67 patients had a positive PCR test for adenovirus (Adv group), while 184 patients had a negative PCR test for adenovirus (Non-Adv group). Among the Non-Adv group patients, no pathogen was detected in 50 patients in all culture assays (unknown pathogen group), while 134 patients were diagnosed with other viruses, bacteria, and combined pathogens (Non-Adv group).

### Comparison of baseline characteristics

Table 1 shows the baseline characteristics of the patients in the Adv and Non-Adv groups at admission. The median age was 21.6 years, and all patients were healthy males prior to the onset of illness. The number of current smokers was significantly higher in the Adv group than in the Non-Adv group (22.2% vs. 5.4%, respectively), with recent smokers (< 30 days) only identified in the Adv group (*n* = 4). A few patients had an underlying disease, such as asthma (*n* = 3), allergic rhinitis (*n* = 3), and pneumothorax (*n* = 2). No differences in the duration of symptoms and the time from symptom onset to admission were observed between the two groups (3.6 ± 1.8 d *vs. *3.2 ± 2.3 d, *P* = 0.224). All patients had clinical symptoms and signs of upper or lower respiratory tract infection. Adv group patients showed more symptomatic instabilities, such as fever, cough, myalgia, headache, and nasal congestion. At admission, initial vital signs and pneumonia severity index (PSI) scores were not significantly different between the two groups.

**Table 1:** Comparisons of baseline characteristics at admission

Characteristics	Adv (*n* = 67)	Non-Adv (*n* = 184)	Total (*n* = 251)	*P* value
Age (year, M[Q_1_-Q_3_])	21.5 [20.0–22.0]	21.6 [20.0–22.0]	21.6 [20.0–22.0]	0.559
Male [*n* (%)]	67 (100)	184 (100)	251 (100)	NA
Smoking [*n* (%)]				< 0.001
Never smoker	45 (67.1)	125 (67.9)	170 (67.7)	
Ex-smoker	7 (10.4)	49 (26.6)	56 (22.3)	
Current smoker	15 (22.4)	10 (5.4)	25 (9.9)	
Recent smoker (< 30 days)	4 (6.0)	0 (0)	4 (1.6)	
BMI (kg/m^2^, *x* ± *s*)	23.6 ± 4.3	23.2 ± 3.3	23.3 ± 3.6	0.664
Underlying condition [*n*(%)]				0.575
Asthma	3 (4.5)	2 (1.1)	5 (2.0)	
Allergic rhinitis	2 (3.0)	1 (0.5)	3 (1.2)	
Pneumothorax	1 (1.5)	1 (0.5)	2 (0.8)	
Symptom duration(Time from symptom onset to admission) (d, *x* ± *s*)	3.6 ± 1.8	3.2 ± 2.3	3.3 ± 2.1	0.224
Symptom and sign [*n*(%)]				
Fever	66 (98.5)	157 (85.4)	223 (88.8)	0.021
Cough	65 (96.9)	157 (85.4)	222 (88.4)	0.025
Myalgia	31 (46.5)	56 (30.4)	87 (34.7)	0.001
Dyspnea(> mMRC scale II)	5 (7.7)	11 (6.1)	16 (6.4)	0.286
Purulent sputum	23 (33.8)	58 (31.7)	81 (32.3)	0.335
Headache	48 (72.2)	96 (52.4)	144 (57.4)	0.014
Nasal congestion/rhinorrhea	44 (65.3)	103 (55.8)	147 (58.6)	0.015
Initial vital signs (*x* ± *s*)				
Systolic blood pressure (mmHg)	125.4 ± 12.5	124.7 ± 13.1	124.9 ± 13.0	0.598
Heart rate (beats/min)	92.5 ± 14.6	91.2 ± 15.2	91.7 ± 14.9	0.335
Respiratory rate (beats/min)	18.5 ± 3.2	18.3 ± 3.8	18.3 ± 3.6	> 0.999
SpO_2_ on room air (%)	97.5 ± 2.3	96.8 ± 2.0	97.0 ± 2.1	0.679
Pneumonia severity index (PSI) score M[Q_1_-Q_3_]	71.0 [61.0–95.0]	75 [60.0–96.0]	74 [60.5–95.9]	0.204

Data are shown as mean ± standard deviation, median [interquartile range] or number (%)

*Adv* Adenovirus, *NA* Not applicable, *mMRC* Modified Medical Research Council, *SpO*_*2*_ Stands for peripheral capillary oxygen

### Laboratory and radiological findings between the Adv and non-Adv groups

We compared the laboratory and radiologic findings between the Adv and Non-Adv groups (Table 2). The percentage of patients having leukopenia and thrombocytopenia was higher in the Adv patients *P* <  0.001), while leukocytosis was more common in the Non-Adv group patients (*P* = 0.035). The levels of infection markers, such as C-reactive protein (CRP) and procalcitonin showed no difference between the two groups. In addition, total bilirubin and creatinine levels showed no significant difference between the two groups.

**Table 2:** Comparisons of laboratory and radiologic parameters between Adv and Non-Adv groups

Variables	Adv (*n* = 67)	Non-Adv (*n* = 184)	Total (*n* = 251)	*P* value
Results of laboratory study				
WBC count (10^9^/L)	6.02 ± 4.15	8.13 ± 4.05	7.57 ± 4.08	0.020
Leukopenia (< 4 × 10^9^/L)	21 (31.3)	10 (5.4)	31 (12.4)	< 0.001
Leukocytosis (> 10 × 10^9^/L)	8 (11.9)	52 (28.0)	60 (23.9)	0.035
Lymphocyte (%)	22.15 ± 8.23	18.62 ± 9.15	19.56 ± 8.90	0.054
Monocyte (%)	12.05 ± 2.72	11.02 ± 3.45	11.49 ± 3.26	0.202
Platelet count (10^9^/L)	136.3 ± 52.7	184.6 ± 63.3	171.7 ± 61.2	< 0.001
Thrombocytopenia (< 150 × 10^9^/L)	22 (32.8)	17 (9.2)	39 (15.5)	< 0.001
Total bilirubin (mg/dl)	0.6 ± 0.3	0.6 ± 0.2	0.6 ± 0.2	0.715
Creatinine (mg/dl)	0.57 ± 0.14	0.64 ± 0.14	0.62 ± 0.14	0.442
C-reactive protein (mg/dl)	5.24 ± 2.94	6.02 ± 3.11	6.00 ± 3.05	0.411
Procalcitonin (ng/ml)	0.04 [0.00–0.08]	0.06 [0.00–0.10]	0.05 [0.00–0.08]	0.635
Results of etiologic study [*n*(%)]				
Unknown pathogen	NA	50 (27.2)	50 (20.0)	NA
Viral etiology[*n*(%)]				< 0.001
Adv	67 (100)	NA	67 (26.7)	NA
Rhinovirus	5 (7.5)	18 (9.8)	23 (9.2)	
Influenza A/B virus	4 (6.0)	22 (12.0)	26 (10.4)	
HMPV	(−)	1 (0.5)	1 (0.4)	
RSV	1 (1.5)	3 (1.6)	4 (1.6)	
Parainfluenza virus	1 (1.5)	3 (1.6)	4 (1.6)	
Bacterial etiology [*n*(%)]				< 0.001
*S. pneumoniae*	3 (4.5)	19 (10.3)	22 (8.8)	
*H. influenzae*	3 (4.5)	12 (6.5)	15 (6.0)	
*M. pneumoniae*	5 (7.5)	8 (4.3)	13 (5.2)	
*K. pneumoniae*	(−)	2 (1.1)	2 (0.8)	
Combined etiologies [*n*(%)]				< 0.001
*S. pneumonia* plus *H.pneumoniae*	7 (10.4)	14 (7.6)	21 (8.4)	
Rhinovirus plus *H.pneumoniae*	(−)	22 (12.0)	22 (8.8)	
Influenza A/B virus plus *S.pneumoniae*	1 (1.5)	6 (3.3)	7 (2.8)	
Influenza A/B virus plus *H.influenzae*	(−)	2 (1.1)	2 (0.8)	
RSV plus *H.influenzae*	(−)	2 (1.1)	2 (0.8)	
Results of radiologic study				
Dominant pattern [*n*(%)]				< 0.001
GGO	3(4.5)	6 (3.3)	9 (3.6)	
Consolidation	23 (24.3)	103 (56.0)	126 (50.2)	
GGO plus consolidation	41 (61.2)	75 (40.7)	116 (46.2)	
Distribution [*n*(%)]				0.015
Unilateral	56 (83.5)	133 (72.2)	189 (75.3)	
Bilateral	5 (7.5)	10 (5.4)	15 (6.0)	
Multi-lobar (≥ 3 lobes)	6 (9.0)	41 (22.3)	47 (18.7)	
Pleural effusion [*n*(%)]	2 (3.0)	8 (4.3)	10 (4.0)	0.483

Data are shown as mean ± standard deviation, median [interquartile range] or number (%)

*NA* Not available, *Adv* Adenovirus, *RSV* Respiratory syncytial virus, *HMPV* Human metapneumovirus, *GGO* Ground glass opacity, *S. pneumoniae Streptococcus pneumoniae*, *H. influenzae Haemophilus influenzae*, *M. pneumoniae Mycoplasma pneumoniae*, *K. pneumoniae Klebsiella pneumoniae*

Possible causative agents were identified in 100% of the Adv group patients and in 72.8% (134/184) of the Non-Adv group patients. In some instances, Adv group patients had coinfections with viruses, such as rhinovirus (*n* = 5), influenza A virus (*n* = 4), respiratory syncytial virus (*n* = 1), and parainfluenza virus (*n* = 1). Bacteria or combined etiologies were more common in the Non-Adv group patients. Rhinovirus (40/184, 21.7%) was most commonly identified as the pathogen in the Non-Adv group patients. The most common bacterial pathogens were *Streptococcus pneumoniae* in the Adv group patients (11/67, 16.4%) and *Haemophilus influenzae* in the Non-Adv group patients (52/184, 28.3%).

The most common radiological feature was ground-glass opacity with consolidation in the Adv group and consolidation in the Non-Adv group (*P* <  0.001). Unilateral distribution was dominant in both groups (83.5% *vs. *72.7%), however multilobar (≥ 3 lobes) involvement was more common in the Non-Adv group (9.0 *vs.* 22.3%, *P* = 0.015). The presence of pleural effusion was not significantly different between the two groups.

### Comparisons of fever and response to antipyretics

**Fig. 2:**
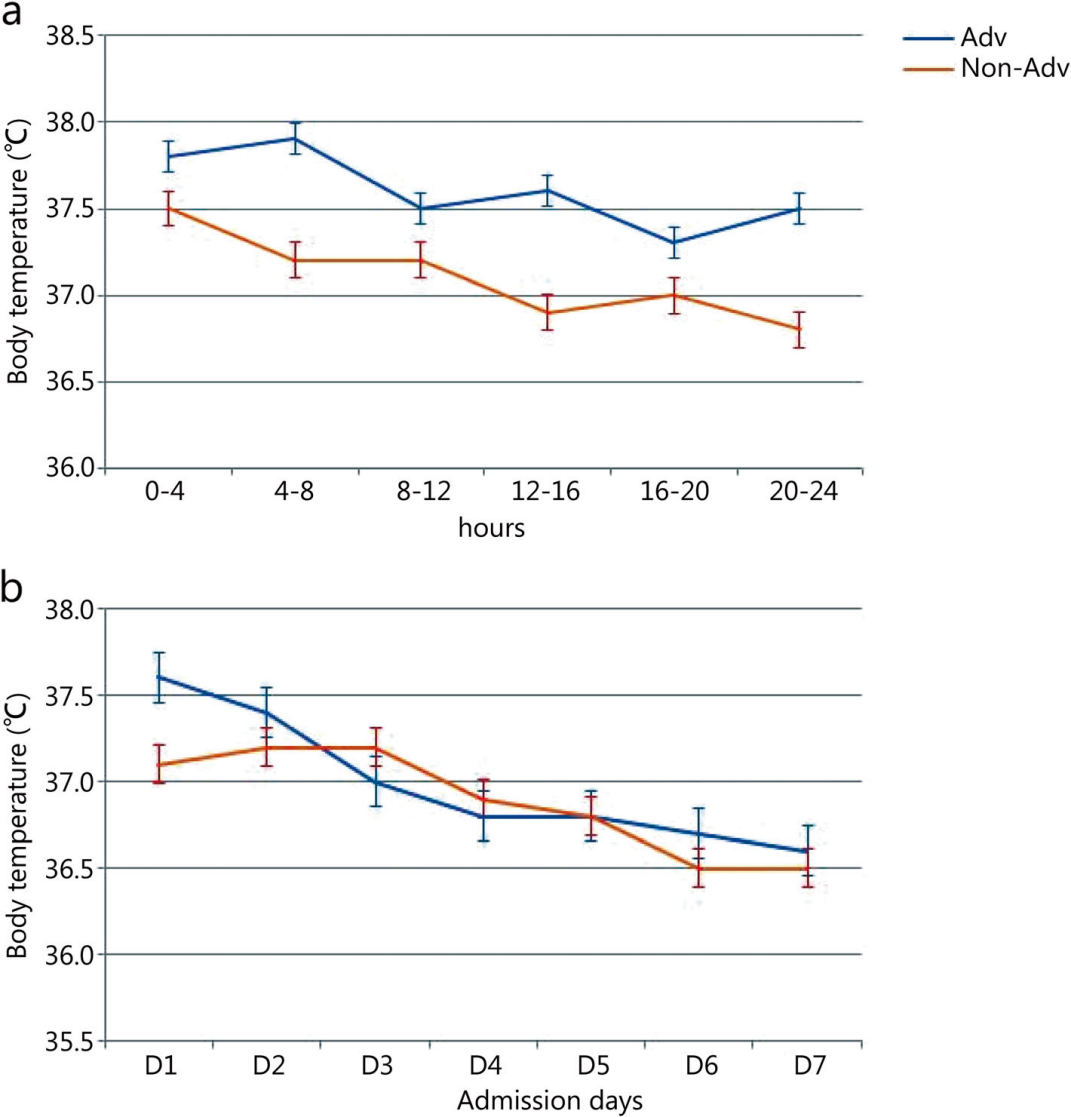
Comparison to the changes in mean body temperature between the Adv-positive and Adv-negative group patients at admission day (**a**) and within 7 days after admission (**b**). **a** Changes in mean temperature in four hours between the Adv and Non-Adv group patients within 24 h at admission (37.6 ± 0.22 *vs.* 37.1 ± 0.25, *P* = 0.005), **b** Comparison of the mean daily temperature between the Adv and Non-Adv group patients for a week (*P* = 0.156)

Figure 2 shows the changes in mean body temperature at admission and during the 7 days after admission between Adv and Non-Adv group patients. In addition, we also compared the fever and response to antipyretic treatment between the patients in the no pathogen group and in the Adv and Non-Adv groups, the results of which are shown in Table 3. In general, Adv group patients had a much longer duration of fever after admission than the Non-Adv patients (3.2 ± 1.6 days *vs.* 1.9 ± 1.2 days, 2.2 ± 1.5 days, *P* = 0.018) and symptom onset (5.8 ± 2.2 days* vs. *3.9 ± 2.5 days, 3.7 ± 2.0 days, *P* = 0.006). To evaluate the degree of fever, we assessed the mean temperature and number of patients to the maximal temperature at admission. The Adv group patients had higher a mean temperature at admission (37.8 ± 0.3 °C* vs.* 37.3 ± 0.3 °C* vs. *37.3 ± 0.2 °C, *P* = 0.005), and more observed instances of a temperature of over 40 and 39 to 40 °C (*P* <  0.001). The Adv group patients took longer to attain the maximal fall in body temperature than the Non-Adv and unknown pathogen group patients at admission (10.2 ± 5.6 *vs.* 8.0 ± 4.5 *vs.* 8.6 ± 5.5, *P* = 0.015).

**Table 3:** Characteristics of fever and response to antipyretics between Adv, Non-Adv, and unknown pathogen group

Variables	Adv group (*n* = 67)	Non-Adv group (*n* = 134)	Unknown pathogen group (*n* = 50)	*P* value
Duration of fever after admission (d,, *x* ± *s*)	3.2 ± 1.6	1.9 ± 1.2	2.2 ± 1.5	0.018
Duration of fever after symptom onset (d, *x* ± *s*)	5.8 ± 2.2	3.9 ± 2.5	3.7 ± 2.0	0.006
Mean temperature at admission day (°C, *x* ± *s*)	37.8 ± 0.3	37.3 ± 0.3	37.3 ± 0.2	0.005
Numbers of patients to maximal temperature at admission [*n*(%)]				< 0.001
Over 40 °C	10 (14.9)	3 (2.2)	2 (4.0)	
39–40 °C	24 (35.8)	5 (3.7)	3 (6.0)	
Time of maximal falls in temperature at admission (h, *x* ± *s*)	10.2 ± 5.6	8.0 ± 4.5	8.6 ± 5.5	0.015
Mean change of temperature at 1 h after administrated antipyretics (°C, *x* ± *s*)	1.1 ± 0.7	1.2 ± 0.6	1.0 ± 0.7	0.645
Responsiveness to antipyretics at admission [*n*(%)]				< 0.001
Complete response	30 (44.8)	84 (62.7)	38 (76.0)	
Partial response	25 (37.3)	48 (35.8)	12 (24.0)	
No response	12 (17.9)	2 (1.5)	(−)	

Data are shown as mean ± standard deviation, median [interquartile range] or number (%)

Approximately 18% of Adv group patients had no response to antipyretic treatment, which represented a higher proportion compared with that observed in the Non-Adv or unknown pathogen group patients (*P* < 0.001). However, the proportion of complete response to antipyretic treatment was comparatively lower in patients in the Adv group than that observed in the Non-Adv or unknown pathogen groups.

### Comparison of clinicolaboratory findings between the Adv and non-Adv groups in patients who were unresponsive to initial antibiotics treatment

The physician suspected the patient of having atypical pathogens when they had persistent or deteriorating symptoms or signs despite treatment with appropriate empirical antibiotics for 2–3 days. Thus, we compared the clinicolaboratory findings between Adv and Non-Adv group patients who were unresponsive to the initial antibiotics treatment (Table 4). The number of patients who did not a response to initial antibiotics treatment was 47 and 50 in the Adv and Non-Adv groups, respectively. The percentage of patients having leukocytosis and monocytopenia was higher in the Adv patients, although there was no significant difference in white blood cell and platelet counts between the two groups. Leukopenia and thrombocytopenia, which were a showed a significant difference in all study patients, showed no difference in patients with unresponsiveness to initial antibiotics treatment (*P* = 0.720, *P* = 0.733, respectively).

**Table 4:** Comparison of clinico-laboratory findings between Adv and Non-Adv patients in whom unresponsive to initial antibiotics treatment

Variables	Adv group (*n* = 47)	Non-Adv group (*n* = 50)	Total (*n* = 97)	*P* value
WBC count (10^9^/L, *x* ± *s*)	5.89 ± 3.75	6.05 ± 3.54	5.95 ± 3.66	0.720
Leukopenia (< 4 × 10^9^/L) [*n*(%)]	21 (44.7)	20 (40.0)	41 (42.3)	0.435
Leukocytosis (> 10 × 10^9^/L) [*n*(%)]	8 (17.0)	15 (30.0)	23 (23.7)	0.015
Lymphocyte (%, *x* ± *s*)	22.15 ± 8.23	18.62 ± 9.15	19.56 ± 8.90	0.054
Monocyte (%, *x* ± *s*)	8.05 ± 3.72	11.02 ± 3.45	9.65 ± 3.56	0.002
Monocytopenia (< 150/μl) [*n*(%)]	8 (17.0)	2(4.0)	10 (10.3)	0.005
Platelet count (10^9^/L, *x* ± *s*)	128.5 ± 62.5	125.5 ± 59.5	126.7 ± 61.5	0.335
Thrombocytopenia (< 150 × 10^9^/L) [*n*(%)]	25 (53.2)	26 (52.0)	51 (52.6)	0.736
Responsiveness to antipyretics at admission [*n*(%)]				0.045
Complete response	4 (8.5)	5 (10.0)	9 (9.3)	
Partial response	31 (66.0)	40 (80.0)	71 (73.2)	
No response	12 (25.5)	5 (10.0)	17 (17.5)	
Numbers of maximal temperature at admission [*n*(%)]				0.003
Over 40 °C	8 (17.0)	3 (6.0)	11 (11.3)	
39–40 °C	21 (44.7)	6 (12.0)	27 (27.8)	
Mean temperature at admission day (°C, *x* ± *s*)	37.8 ± 0.3	37.3 ± 0.2	37.5 ± 0.2	0.005
Duration of fever after admission (d, *x* ± *s*)	3.3 ± 1.5	2.8 ± 1.6	3.0 ± 1.5	0.156

Data are shown as mean ± standard deviation or number (%)

A greater number of Adv group patients exhibited no response to antipyretic treatment compared with the Non-Adv group patients (25.5% *vs. *10.0%, *P* = 0.045) as well as the number of patients to reach over 40 °C and 39 to 40 °C (*P* = 0.003). In addition, the Adv group patients had a higher mean temperature at admission than the Non-Adv group patients (37.8 ± 0.3* vs.* 37.3 ± 0.2, *P* = 0.005).

Table 5 compares the clinicolaboratory variables between the combined Adv (cAdv), Non-Adv, and only Adv identified pathogen (OAIP) group patients. Compared to the cAdv and Non-Adv patients, more patients in the OAIP group exhibited the following characteristics: were currently smoking; had leukopenia, lymphopenia, monocytopenia, and thrombocytopenia; exhibited a longer duration of fever after symptom onset; had a higher maximal temperature at admission (over 40 °C and 39–40 °C); and exhibited no response to antipyretics at admission.

**Table 5:** Comparison of clinico-laboratory variables between combined Adv (cAdv), Non-Adv, and only Adv identified pathogen (OAIP) groups

Variables	cAdv group^a^ (*n* = 30)	Non-Adv group (*n* = 184)	OAIP group^b^ (*n* = 37)	*P* value
Age (year, M[Q_1_-Q_3_])	21.5 [20.0–22.0]	21.6 [20.0–22.0]	21.5 [20.0–22.0]	0.672
Smoking [*n*(%)]				< 0.001
Never smoker	21 (70.0)	125 (67.9)	24 (64.9)	
Ex-smoker	3 (10.0)	49 (26.6)	4 (10.8)	
Current smoker	6 (20.0)	10 (5.4)	9 (24.3)	
Recent smoker (< 30 d)	2 (6.7)	0 (0)	2 (5.4)	
Symptom duration(Time from symptom onset to admission) (d, *x* ± *s*)	3.6 ± 1.5	3.2 ± 2.3	3.6 ± 2.0	0.154
Symptom and sign [*n*(%)]				
Fever	30 (100.0)	157 (85.4)	36 (97.3)	0.062
Cough	29 (96.7)	157 (85.4)	36 (97.3)	0.145
Myalgia	14 (46.7)	56 (30.4)	17 (45.9)	0.264
Dyspnea (> mMRC scale II)	3 (10.0)	11 (6.1)	2 (5.4)	0.510
Purulent sputum	12 (40.0)	58 (31.7)	11 (29.7)	0.126
Headache	20 (66.7)	96 (52.4)	28 (75.7)	0.089
Nasal congestion/rhinorrhea	20 (66.7)	103 (55.8)	24 (64.9)	0.423
WBC count (10^9^/L, *x* ± *s*)	7.54 ± 4.28	8.13 ± 4.05	4.50 ± 4.02	0.045
Leukopenia (< 4 × 10^9^/L) [*n*(%)]	6 (20.0)	10 (5.4)	15 (40.5)	< 0.001
Leukocytosis (> 10 × 10^9^/L) [*n*(%)]	5 (16.7)	52 (28.0)	3 (8.1)	< 0.001
Lymphocyte (%, *x* ± *s*)	23.84 ± 8.74	18.62 ± 9.15	20.46 ± 7.72	0.049
Monocyte (%, *x* ± *s*)	14.75 ± 1.69	11.02 ± 3.45	9.35 ± 3.75	0.002
Platelet count (10^9^/L, *x* ± *s*)	141.4 ± 45.7	184.6 ± 63.3	131.2 ± 59.7	< 0.001
Thrombocytopenia (< 150 × 10^9^/L) [*n*(%)]	7 (23.3)	17 (9.2)	15 (40.5)	< 0.001
Duration of fever after admission (d, *x* ± *s*)	2.9 ± 1.8	2.0 ± 1.3	3.5 ± 1.4	0.121
Duration of fever after symptom onset (d, *x* ± *s*)	5.6 ± 2.2	3.8 ± 2.4	6.0 ± 2.2	0.015
Mean temperature at admission day (°C, *x* ± *s*)	37.8 ± 0.3	37.3 ± 0.3	37.7 ± 0.4	0.223
Numbers of maximal temperature at admission [*n*(%)]				
Over 40 °C	4 (13.3)	5 (2.7)	6 (16.2)	0.016
39–40 °C	10 (33.3)	8 (4.3)	14 (37.8)	0.024
Responsiveness to antipyretics at admission [*n*(%)]				< 0.001
Complete response	16 (53.3)	122 (66.3)	14 (37.8)	
Partial response	12 (40.0)	60 (32.6)	13 (35.1)	
No response	2 (6.7)	2 (1.1)	10 (27.1)	

Data are shown as mean ± standard deviation, median [interquartile range] or number (%)

*Adv* Adenovirus, *mMRC* Modified Medical Research Council, *CAP* Community-acquired pneumonia

^a^cAdv defined that CAP patients with adenovirus combined other virus or bacteria as identified pathogen; ^b^OAIP defined that CAP patients with adenovirus as the only identified pathogen

### Comparison of treatment outcome

All patients received empirical antibiotic treatment (Table 6) as follows: a 3rd generation cephalosporin plus azithromycin was the most common regimen (*n* = 243, 96.8%), followed by piperacillin/tazobactam plus respiratory quinolone (*n* = 5, 2.0%). The change in antibiotics treatment regimen was more frequent in the Adv group patients than in the Non-Adv patients (70.1% *vs.* 27.2%, *P* = 0.024). The duration of antibiotic treatment was not significantly different between the two groups. In our study, we did not evaluate the administration of cidofovir or adjuvant intravenous immunoglobulin (IVIG). In addition, there were no patients who received mechanical ventilation or extracorporeal membrane oxygenation support.

**Table 6:** Comparisons of treatment outcomes between Adv and Non-Adv group

Variables	Adv (*n* = 67)	Non-Adv (*n* = 184)	Total (*n* = 251)	*P* value
Initial antibiotics regimen				0.781
3rd cephalosporin plus azithromycin	65 (97.0)	178 (96.7)	243 (96.8)	
Respiratory quinolone	(−)	1 (0.5)	1 (0.4)	
Piperacillin/tazobactam plus quinolone	1 (1.5)	4 (2.2)	5 (2.0)	
Piperacillin/tazobactam	(−)	1 (0.5)	1 (0.4)	
Carbapenem	1 (1.5)	(−)	1 (0.4)	
Treatment regimen change (Antibiotics escalation)	47 (70.1)	50 (27.2)	97 (38.6)	0.024
Duration of antibiotics use, day	12.32 ± 2.76	11.64 ± 2.89	11.85 ± 2.83	0.114
Mean antipyretics dose at admission, gram	5.52 [3.45–6.91]	4.30 [3.14–6.55]	4.85 [3.21–6.75]	0.032
Duration of antipyretics use, days	10.5 ± 2.7	10.6 ± 3.1	10.6 ± 3.0	0.892
Adverse event after antipyretics use				0.005
Hypotension	10 (14.9)	4 (2.2)	14 (5.6)	
GI trouble	6 (9.0)	2 (1.1)	8 (3.2)	
Skin rash	1 (1.5)	(−)	1 (0.4)	
Elevated liver enzyme	4 (6.0)	2 (1.1)	6 (2.4)	
Length of hospital stay	15.0 ± 2.3	14.8 ± 2.1	14.9 ± 2.2	0.407
Time from admission to improvement of discomfort, day	4.3 ± 2.8	2.9 ± 1.8	3.2 ± 2.0	0.034
In-hospital mortality	0 (0)	0 (0)	0 (0)	> 0.999

Data are shown as mean ± standard deviation, median [interquartile range] or number (%)

*Adv* Adenovirus; *GI* Gastrointestinal

At admission, the mean dose of antipyretics administered was higher in the Adv group patients than in the Non-Adv group patients (5.52 *vs.* 4.30 g, *P* = 0.032), although the overall duration of antipyretics was not significantly different between the two groups. In this study, we identified adverse events after antipyretics administration, such as hypotension, gastrointestinal trouble, skin rash, and elevated liver enzyme, which were commonly observed in the Adv group patients (*P* = 0.005).

The time to overall clinical stabilization from admission was significantly longer in the Adv group patients than in the Non-Adv group patients (4.3 ± 2.8 d *vs.* 2.9 ± 1.8 d, *P* = 0.034). In addition, the length of hospital was not significantly different between the two groups, and no patient died in our study.

## Discussion

In our study, we described the clinical characteristics of Adv-positive community-acquired pneumonia in immunocompetent adult patients. The most important findings were that Adv group patients had a longer duration of fever after symptom onset and admission, a higher mean body temperature at admission, a higher number of patients with a body temperature over 39 °C at admission, a longer duration of the maximal fall in body temperature at admission, and higher rate of no response to antipyretic treatment at admission compared to the Non-Adv group patients. In addition, the instances of leukopenia and thrombocytopenia were greater in the Adv group patients than in the Non-Adv group patients, although in patients who were unresponsive to the initial antibiotics treatment, there was no difference between the two groups. Some epidemiological studies of Adv in South Korea military trainees and personnel have been performed. Yoo et al. [15] reported that adenovirus was identified in 33.0% of all specimens in febrile respiratory illness (FRI) or pneumonia patients. This study was of reviewed military patients with FRI or pneumonia who were tested for respiratory viruses from October 2014 to May 2016. The proportion of patients with pneumonia and the hospitalization rate did not differ between those with and without adenovirus infection. However, adenovirus-infected patients had a significantly higher risk of requiring intensive care or mechanical ventilator support. These notable findings indicate that adenovirus infection has been occasionally associated with mortality and morbidity with loss in combat strength and an increase in the cost of care.

To date, little data has been reported on the characteristics of fever during Adv infection, especially in immunocompetent patients. In this study, we compared the characteristics of fever in Adv and Non-Adv group patients. The Adv group patients had a longer duration of fever and a higher proportion of peak body temperature than patients infected with other various viral respiratory pathogens or unknown pathogens. Similarly, Ho et al [22] observed that viral mono-pathogen patients had a higher mean body temperature than bacterial mono-pathogen patients. In addition, dual-pathogen patients, such as *S. pneumoniae* with either influenza A or B, had a higher mean body temperature, although not significantly different from the respective mono-pathogens. However, there is still a lack of detailed data on the specific pathogen-related clinical characteristics, especially fever, in immunocompetent patients. Thus, our data is more likely to aid physicians in determining further diagnostic or therapeutic considerations at the time of admission.

We also evaluated the response to antipyretic treatment between the Adv and Non-Adv groups. Our data showed that the Adv group patients exhibited a higher proportion of individuals with no response to antipyretic treatment compared with the Non-Adv or unknown pathogen group patients. Weisse et al. [23] evaluated the effect of acetaminophen on fever in bacterial vs. viral infections in 100 children. They concluded that there is no correlation between a fever response to acetaminophen and the etiology of the fever, revealing no usefulness in the response to antipyretic treatment in predicting etiologies of pneumonia. However, our data suggest that there may be a difference in the antipyretic response to Adv compared to other etiologies, and this is the first such data reported for immunocompetent adults.

Our study had several limitations. First, our study was a retrospective design in a single-center, so confounding variables, such as antibiotics regimen or inconsistent timing of antipyretics administration, may have possibly affected the clinical course of fever or response to antipyretic treatment. Second, our study had few reflected unmeasurable variables, such as the genotype of adenovirus, so the difference in the severity of illness between the Adv and Non-Adv groups may have led to the difference in the characteristics of fever and its response to antipyretics. Third, we conducted our study in a military hospital, so our cohort was not representative of the general population, since the military environment has different characteristics, such as living environment, nutrition/immune status, and mode of pathogen spread. Fourth, there was selection bias in that all patients admitted with CAP could not have respiratory PCR tests performed; the examination was limited to an upper respiratory tract, and Adv serotype or viral burden analyses were not performed. Fifth, several potentially relevant pathogens were identified in the study patients. Thus, it is uncertain whether adenovirus was the “leading” pathogen in all patients with multiple pathogens that included adenovirus. Additionally, it is uncertain whether the patients with unknown pathogens were not infected with pathogens.

## Conclusions

To the best of our knowledge, this is the first study to analyze the characteristics of fever and response to antipyretic therapy in immunocompetent adult patients with adenovirus-infected CAP. Patients in the Adv group had some clinical characteristics that were significantly different from those in the Non-Adv group, including a longer duration of fever, a high fever (over 39 °C), and a higher proportion of no response to antipyretic treatment at admission.

## Data Availability

All data generated or analyzed during the present study are included in this published article.

## Abbreviations

Adv: Adenovirus
cAdv: Combined Adv
CAP: Community-acquired pneumonia
CPR: C-reactive protein
FRI: Febrile respiratory illness
HRCT: High resolution computed tomography
OAIP: Only Adv identified the pathogen
PCR: Polymerase chain reaction

## References

[1] Campbell SJ, Kynyk JA, Davis JA. Disseminated adenovirus infection causing severe ARDS. BMJ Case Rep. 2017;2017. 10.1136/bcr-2016-217524. 10.1136/bcr-2016-217524 PMC5256538 28096226

[2] Lynch JP, Fishbein M, Echavarria M (2011). Adenovirus. Semin Respir Crit Care Med.

[3] Lynch JP, Kajon AE (2016). Adenovirus: epidemiology, global spread of novel serotypes, and advances in treatment and prevention. Semin Respir Crit Care Med.

[4] Yatsyshina SB, Samchuk VV, Vasilyev VV, Ageeva MR, Vorobyeva NS, Savochkina YA (2014). Adenovirus pneumonia with a fatal outcome in adults. Ter Arkh.

[5] Chen HL, Chiou SS, Hsiao HP, Ke GM, Lin YC, Lin KH (2004). Respiratory adenoviral infections in children: a study of hospitalized cases in southern Taiwan in 2001-2002. J Trop Pediatr.

[6] Cheng JL, Peng CC, Chiu NC, Weng LC, Chiu YY, Chang L (2017). Risk factor analysis and molecular epidemiology of respiratory adenovirus infections among children in northern Taiwan, 2009-2013. J Microbiol Immunol Infect.

[7] Clark TW, Fleet DH, Wiselka MJ (2011). Severe community-acquired adenovirus pneumonia in an immunocompetent 44-year-old woman: a case report and review of the literature. J Med Case Rep.

[8] Hakim FA, Tleyjeh IM (2008). Severe adenovirus pneumonia in immunocompetent adults: a case report and review of the literature. Eur J Clin Microbiol Infect Dis.

[9] Hung KH, Lin LH (2015). Adenovirus pneumonia complicated with acute respiratory distress syndrome: a case report. Medicine (Baltimore).

[10] Kajon AE, Hang J, Hawksworth A, Metzgar D, Hage E, Hansen CJ (2015). Molecular epidemiology of adenovirus type 21 respiratory strains isolated from US military trainees (1996-2014). J Infect Dis.

[11] Van Kerkhove MD, Cooper MJ, Cost AA, Sanchez JL, Riley S (2015). Risk factors for severe outcomes among members of the United States military hospitalized with pneumonia and influenza, 2000-2012. Vaccine.

[12] Jeon K, Kang CI, Yoon CH, Lee DJ, Kim CH, Chung YS (2007). High isolation rate of adenovirus serotype 7 from south Korean military recruits with mild acute respiratory disease. Eur J Clin Microbiol Infect Dis.

[13] Park JY, Kim BJ, Lee EJ, Park KS, Park HS, Jung SS (2017). Clinical features and courses of adenovirus pneumonia in healthy young adults during an outbreak among Korean military personnel. PLoS One.

[14] Park PK, Cannon JW, Ye W, Blackbourne LH, Holcomb JB, Beninati W (2016). Incidence, risk factors, and mortality associated with acute respiratory distress syndrome in combat casualty care. J Trauma Acute Care Surg.

[15] Yoo H, Gu SH, Jung J, Song DH, Yoon C, Hong DJ (2017). Febrile respiratory illness associated with human adenovirus type 55 in South Korea military, 2014-2016. Emerg Infect Dis.

[16] Yoon H, Jhun BW, Kim H, Yoo H, Park SB (2017). Characteristics of adenovirus pneumonia in Korean military personnel, 2012-2016. J Korean Med Sci.

[17] Hwang SM, Park DE, Yang YI, Park SJ, Lee HK, Kim MJ (2013). Outbreak of febrile respiratory illness caused by adenovirus at a south Korean military training facility: clinical and radiological characteristics of adenovirus pneumonia. Jpn J Infect Dis.

[18] Cha MJ, Chung MJ, Lee KS, Kim TJ, Kim TS, Chong S (2016). Clinical features and radiological findings of adenovirus pneumonia associated with progression to acute respiratory distress syndrome: a single center study in 19 adult patients. Korean J Radiol.

[19] Yoon H, Jhun BW, Kim SJ, Kim K (2016). Clinical characteristics and factors predicting respiratory failure in adenovirus pneumonia. Respirology.

[20] Mandell LA, Wunderink RG, Anzueto A, Bartlett JG, Campbell GD, Dean NC (2007). Infectious Diseases Society of America/American Thoracic Society consensus guidelines on the management of community-acquired pneumonia in adults. Clin Infect Dis.

[21] Song JH, Jung KS, Kang MW, Kim DJ, Pai H, Suh GY (2009). Treatment guidelines for community-acquired pneumonia in Korea: an evidence-based approach to appropriate antimicrobial therapy. Tuberc Respir Dis.

[22] Ho ZJ, Zhao X, Cook AR, Loh JP, Ng SH, Tan BH (2015). Clinical differences between respiratory viral and bacterial mono- and dual pathogen detected among Singapore military servicemen with febrile respiratory illness. Influenza Other Respir Viruses.

[23] Weisse ME, Miller G, Brien JH (1987). Fever response to acetaminophen in viral vs. bacterial infections. Pediatr Infect Dis J.

